# Presumptive Acute Post‐Hypoxic Myoclonus Following Return of Spontaneous Circulation in a Cat and a Dog

**DOI:** 10.1111/vec.70140

**Published:** 2026-07-26

**Authors:** Theofanis Liatis, Alison Robertson, Alberta De Stefani

**Affiliations:** ^1^ Queen Mother Hospital For Animals, Royal Veterinary College University of London Hatfield UK

**Keywords:** cardiopulmonary arrest, global brain hypoxia, global brain ischemia, movement disorder, post‐anoxic myoclonus

## Abstract

**Objective:**

To describe the clinical presentation of presumptive acute post‐hypoxic myoclonus in a cat and a dog after cardiopulmonary arrest (CPA). In people, acute post‐hypoxic myoclonus is a transient focal, multifocal, or generalized rhythmic myoclonus that results from global hypoxic–ischemic brain injury and may be cortical or subcortical in origin, specifically brainstem‐generated. Post‐hypoxic myoclonus typically presents within 48 h of CPA while the patient is still comatose. Although this neurologic sign has historically been associated with a poorer prognosis, recent studies suggest that evaluation of its clinical characterization in conjunction with electroencephalographic features should be considered.

**Case Series Summary:**

We describe the cases of one cat and one dog that suffered CPA, underwent CPR, and experienced return of spontaneous circulation. Both cases developed presumed acute post‐hypoxic myoclonus within 24 h of resuscitation, did not respond to antiseizure and sedative drugs, and were subsequently euthanized.

**New or Unique Information Provided:**

Acute post‐hypoxic myoclonus can occur in cats and dogs after CPA and successful resuscitation.

**Abbreviations**
CPA: cardiopulmonary arrest
CRI: constant rate infusion
EEG: electroencephalography
EMG: electromyography
GHIBI: global hypoxic–ischemic brain injury
MRI: magnetic resonance imaging
MSE: myoclonic status epilepticus
PHM: post‐hypoxic myoclonus
PLR: pupillary light reflex
RI: reference interval
ROSC: return of spontaneous circulation
VOR: vestibulo‐ocular reflex

## 1 Introduction

Myoclonus is a sudden, brief, involuntary, shock‐like movement of an affected body part. It is most commonly a result of muscular contraction (positive) or, less frequently, inhibition of muscle tone (negative or asterixis) [1, 2]. Clinically, myoclonus can involve different body parts individually (focal), contiguously (segmental), globally synchronously (generalized), or asynchronously (multifocal) [3]. Myoclonus can be episodic or repetitive; when repetitive, the jerks are usually irregular (arrhythmic); in some cases, however, repetitive myoclonus can present as regular, in which case it may mimic tremors [2, 3]. Myoclonus can occur at rest, during action, or while maintaining a posture; in some cases, it can be provoked by a stimulus (reflex myoclonus) [2].

Acute post‐hypoxic myoclonus (PHM) is a transient, rhythmic, or irregular myoclonus of cortical or subcortical origin that results from global hypoxic–ischemic brain injury (GHIBI) and typically presents within 48 h of cardiopulmonary arrest (CPA) while the patient is still comatose [4, 5, 6, 7]. Acute PHM can have variable phenotypes. Three distinct clinical phenotypes have recently been identified in people: type 1 (distal, asynchronous, variable), type 2 (axial or axial and distal, asynchronous, variable), and type 3 (axial, synchronous, stereotyped) [8]. Electroencephalography (EEG) is crucial to determine whether a myoclonus is cortical or subcortical in origin [4, 5, 6, 7].

In people, acute PHM has historically been associated with a poor prognosis and was used to guide decisions regarding withdrawal of life support, although this has been under debate in recent literature. Generalized seizures or focal, self‐limiting, brief seizures occurring after GHIBI have been reported in populations of hospitalized or experimental dogs, respectively [9, 10, 11]. However, acute PHM has not been reported in dogs or cats and, more interestingly, has not been considered in the prognostic indicators for survival after CPR in cats and dogs [12]. To the authors’ knowledge, this is the first report to describe presumptive acute PHM in a cat and a dog after return of spontaneous circulation (ROSC) following CPA.

## 2 Case Summary

### 2.1 Case 1

A 10‐year‐old neutered male British Shorthair cat was presented to the hospital due to peracute, nonprogressive neurologic signs after CPR and ROSC. The cat had undergone general anesthesia at the referring veterinarian for rhinoscopy and thoracic radiography to investigate upper airway disease. Thoracic radiographs had been unremarkable. Computed tomography of the head was subsequently performed to investigate the cat's chronic upper respiratory tract signs, which were present before CPA. A moderate amount of soft tissue‐attenuating material was identified within the ventral aspect of the nasal cavities, in addition to mild osteolysis of the ventral nasal turbinates and mild thickening of the mucosal lining at the nasopharynx, changes consistent with rhinitis. While anesthetized for computed tomography, the cat experienced CPA. CPR was performed, and ROSC was achieved on the day of referral.

On presentation to the hospital 3 h after ROSC, a neurologic examination revealed stuporous mentation with decerebrate rigidity, tetraplegia, and present reaction to nociception testing in all four limbs. The cat had absent menace response and palpebral reflexes; decreased pupillary light reflex (PLR) bilaterally, vestibulo‐ocular reflex (VOR), and gag reflex; and intact spinal reflexes. Intermittent, apparently involuntary muscle contractions were noted. Repeat neurologic assessment 16 h after CPA revealed episodic, spontaneous, generalized myoclonus with an irregular rhythm, often more prominent on the pelvic limbs in lateral recumbency (Video 1). Neuroanatomic localization was consistent with diffuse forebrain and brainstem lesions. A presumptive diagnosis of GHIBI was made, and the presence of acute PHM was suspected.

Hematology revealed marked leukocytosis (47.07 × 10^9^/L; reference interval [RI]: 5.50–19.50 × 10^9^/L) with neutrophilia (44.25 × 10^9^/L; RI: 2.50–12.50 × 10^9^/L). Serum biochemistry panel revealed hyperglobulinemia (52.4 g/L; RI: 25–45 g/L [5.2 g/dL; RI 2.5–4.5 g/dL]), increased BUN concentration (10.1 mmol/L; RI: 2.5–9.9 mmol/L [28.3 mg/dL; RI 7–27.7 mg/dL]), increased alanine aminotransferase activity (223 U/L; RI: 5–60 U/L), and increased creatine kinase activity (1278 U/L; RI: 57–574 U/L). Venous blood gas analysis revealed mild respiratory acidosis (PvCO_2_ 6.73 kPa [50.5 mm Hg]; RI: 4.93–6.27 kPa [37.0–47.0 mm Hg]), moderate hypokalemia (2.9 mmol/L; RI: 3.6–4.6 mmol/L), and mild hyperglycemia (8.1 mmol/L [145.8 mg/dL]; RI: 4.7–7.3 [84.6–131.4 mg/dL]). Point‐of‐care ultrasound showed intermittent B‐lines in the mid‐thorax bilaterally and an otherwise unremarkable thorax and abdomen. These findings were consistent, although not pathognomonic, for pulmonary infiltrates, such as pulmonary contusions from chest compressions during CPR.

Upon presentation, treatment had been initiated with hyperosmolar agents including 7.2% NaCl^1^ (1 mL/kg) and 20% mannitol^2^ (0.5 g/kg, IV), as well as midazolam^3^ (0.3 mg/kg/h, IV, constant rate infusion [CRI]) and Hartmann's^4^ IV fluid therapy (2 mL/kg/h, CRI) with potassium chloride^5^ supplementation (60 mEq/L). After the myoclonus was noted, anticonvulsant drugs were added, including levetiracetam^6^ (60 mg/kg, IV bolus, followed by 30 mg/kg, IV, q 8 h), ketamine^7^ (0.3–0.5 mg/kg/h, IV, CRI), and dexmedetomidine^8^ (0.4–0.5 µg/kg/h, IV, CRI).

The cat became comatose, developing an abnormal respiratory pattern and an absent gag reflex. Venous blood gas revealed persistent, mild respiratory acidosis (PvCO_2_ 6.73 kPa [50.5 mm Hg]; RI: 4.93–6.27 kPa [37–47 mm Hg]). Due to the cat's lack of gag reflex, declining mentation, abnormal breathing pattern, and persistent hypercapnia, the decision was made to intubate. Boluses of midazolam^3^ (0.3 mg/kg, IV, once), ketamine^7^ (0.5 mg/kg, IV, once), and propofol^9^ (0.5 mg/kg, IV, once) were administered for intubation, after which the cat was apneic. Manual positive‐pressure ventilation was performed for 1 h, but the cat remained apneic; therefore, mechanical ventilation^10^ was initiated with volume‐control ventilation. Over 14 h, the sedative medications (midazolam^3^ CRI, ketamine^7^ CRI, and dexmedetomidine^8^ CRI) were reduced and discontinued, and after 24 h, the cat was successfully weaned from mechanical ventilation. Antiepileptic medication was continued, consisting of levetiracetam^6^ (30 mg/kg, IV, q 8 h).

The cat's mentation initially improved to being severely obtunded, and it was intermittently lifting its head; however, the cat became comatose again with no neurologic improvement 48 h after weaning from mechanical ventilation. The owners elected humane euthanasia for poor prognosis. Myoclonus was first noted 16 h following CPA, before the initiation of ketamine, dexmedetomidine, and levetiracetam, and persisted despite anticonvulsants from that time until the time of euthanasia.

### 2.2 Case 2

A 3‐year‐old neutered male Springer Spaniel dog was presented to the hospital for acute, progressive lethargy, anorexia, vomiting, tachycardia, and pleural effusion. Upon presentation, the dog was obtunded and nonambulatory. The heart rate was 350/min, and ECG analysis revealed supraventricular tachycardia. Point‐of‐care ultrasound revealed a small volume of pleural effusion and occasional B‐lines in the middle of the right hemithorax. There was no pericardial or abdominal effusion noted.

Treatment for the supraventricular tachycardia was attempted with incremental doses of lidocaine^11^ (total 8 mg/kg, IV, followed by 50–70 µg/kg/min, IV, CRI). Diltiazem^12^ was then administered in incremental doses (total 0.3 mg/kg, IV). Magnesium^13^ (0.5–0.7 mEq/kg/day, IV, CRI) was administered, followed by amiodarone^14^ (2 mg/kg, IV bolus, followed by 0.4–0.8 mg/kg/h, CRI). The supraventricular tachycardia was refractory to antiarrhythmic treatment.

The next day, electrical cardioversion was attempted. The dog was anesthetized with methadone^15^ (0.1 mg/kg, IV, once), midazolam^3^ (0.35 mg/kg, IV, once), and propofol^9^ (4.8 mg/kg, IV, once). External cardioversion was performed with a cardioverter‐defibrillator^16^ attached to pacing pads on the dog's thorax. Immediately after the first shock was delivered, the dog suffered CPA.

CPR was commenced, and reversals were administered with flumazenil^17^ (0.01 mg/kg, IV, once) and naloxone^18^ (0.04 mg/kg, IV, once). After 2 min of chest compressions, the ECG rhythm in the first planned intercycle pause was pulseless electrical activity. After 4 min of CPR, ventricular fibrillation was observed in the second, planned, intercycle pause. External defibrillation was performed a total of four times at 2‐min intervals. The cardiac rhythm then converted to asystole after ∼12 min of CPR. A bolus of atropine^19^ (0.05 mg/kg, IV, once) and amiodarone^14^ (2 mg/kg, IV, once) was administered. ROSC was achieved after 20 min of CPR. The dog subsequently experienced ventricular tachycardia, and a dose of lidocaine^11^ (2 mg/kg, IV, once) was administered. After this, there were occasional ventricular premature complexes in a background of normal sinus rhythm.

Fourteen hours after experiencing CPA, the dog was reported to manifest involuntary muscle contractions at rest without external stimulation. Neurologic examination revealed stuporous mentation, tetraplegia, and present reaction to nociception testing in all four limbs. The dog had an absent menace response; present palpebral reflexes and bilateral miosis; decreased bilateral PLR, gag reflex, and VOR; and intact spinal reflexes. An episodic, spontaneous generalized myoclonus, with irregular rhythm, often more prominent in the pelvic limbs, was manifested in lateral recumbency (Video 1). Neuroanatomic localization was consistent with a diffuse forebrain and brainstem lesion. A presumptive diagnosis of GHIBI was made, and the presence of acute PHM was suspected.

Hematology and serum biochemistry panel were within normal limits. C‐reactive protein was increased (85.3 mg/L; RI: 0–10 mg/L). Venous blood gas analysis showed hyperlactatemia (2.6 mmol/L; RI: 0.6–2.5 mmol/L). Cardiac troponin was markedly increased (5.32 µg/L [5.32 ng/mL]; RI: 0–0.11 ng/mL [0–0.11 ng/mL]). Total T4 and thyroid‐stimulating hormone concentrations were within normal limits. Echocardiography after cardioversion revealed bi‐atrial dilation and a hypokinetic left ventricle. Serology for vector‐borne diseases (*Borrelia* spp., *Ehrlichman* spp., *Anaplasma* spp., *Dirofilaria* spp.)^20^ was negative. Blood and urine bacterial and fungal cultures were negative. A clear cause of the dog's presenting cardiac arrhythmia could not be identified, and a tentative diagnosis of immune‐mediated myocarditis was made.

Post‐CPA care included treatment for myocarditis with immunosuppressive doses of dexamethasone^21^ (0.4 mg/kg, IV, q 24 h) and antiarrhythmics including lidocaine^11^ (50–70 µg/kg/min, IV, CRI), magnesium^13^ (0.5–0.7 mEq/kg/day, IV, CRI), amiodarone^14^ (0.4–0.8 mg/kg/h, IV, CRI, followed by 5 mg/kg, PO, q 12 h), diltiazem^12^ (0.1 mg/kg, PO, q 8 h), and sotalol^22^ (2 mg/kg, PO, q 12 h). Anticonvulsants including levetiracetam^6^ (30 mg/kg, IV, q 8 h), sedatives including dexmedetomidine^8^ (0.5–1 µg/kg/h, IV, CRI) and trazodone^23^ (5 mg/kg, PO, q 8 h), and oxygen therapy through nasal cannulae (250 mL/kg/min) were provided.

Although the arrhythmias improved, the dog developed progressive hypoxemia. High‐flow nasal oxygen therapy^24^ was trialed; however, the dog remained hypoxemic and required intubation and mechanical ventilation^10^ 48 h after ROSC. The dog was administered midazolam^3^ (0.3 mg/kg, IV, once) and propofol^9^ (1 mg/kg, IV, once) to facilitate intubation and was started on pressure‐control ventilation. On repeat point‐of‐care thoracic ultrasound, B‐lines were seen in the dog's right cranioventral thorax that had progressed to consolidation of the right and left cranioventral lung regions.

Cytology of an endotracheal wash revealed neutrophilic inflammation with rare intracellular, elongated oval structures, thought possibly to be bacteria. It was suspected the dog had pulmonary contusions secondary to chest compressions and had also developed aspiration pneumonia. Antimicrobials were initiated, consisting of amoxicillin–clavulanic acid^25^ (20 mg/kg, IV, q 8 h). The dog was maintained on mechanical ventilation for just over 48 h and was then successfully weaned onto oxygen therapy provided by nasal cannulae. Thirty‐six hours after weaning from mechanical ventilation, the dog developed worsening hypoxemia and was euthanized due to financial constraints and poor prognosis. Myoclonus had persisted until the time of euthanasia despite anticonvulsant and sedative drugs.

Postmortem histopathologic examination confirmed multifocal neuronal degeneration with satellitosis and marked endothelial hyperplasia with perivascular edema in the brain, consistent with GHIBI. Additionally, hypoxic injury was evident in the liver in the form of multifocal centrilobular hepatocellular degeneration with midzonal hepatocellular regeneration. Myocarditis (lymphoplasmacytic, eosinophilic, and histiocytic) was confirmed. Necrosuppurative and histiocytic bronchointerstitial pneumonia was also present, consistent with aspiration pneumonia.

## 3 Discussion

This case series describes presumptive acute PHM in a cat and a dog after CPA. Although epileptic seizures after ROSC have been reported in dogs and cats in both clinical and experimental settings, acute PHM has not been yet reported [9, 10, 11].

Acute PHM is a consequence of GHIBI and typically presents in people within 48 h of CPA as focal, multifocal, or generalized myoclonus, usually before the patient regains consciousness and lasting for several days [4, 5, 6, 7, 13]. Although it is usually spontaneous, acute PHM can also be stimulus triggered [4]. It is usually a rhythmic myoclonus and is thus thought to be subcortical in origin, more specifically, a brainstem‐generated myoclonus [5, 14]. Acute PHM has historically been considered to originate in the reticular formation of the medulla oblongata and was previously called “reticular reflex myoclonus” [15]. This type of myoclonus is stimulus sensitive and generalized, with initial activation of the muscles innervated by lower brainstem nuclei followed by rostral and caudal recruitment spread [14]. However, in some cases, especially when myoclonus is multifocal, a cortical origin has been considered [5]. Patients with acute generalized PHM can be at risk of myoclonic status epilepticus (MSE), which is continuous (>30 min) or repeated generalized myoclonus and is associated with a grave prognosis [5]. The reported response to treatment in patients with MSE associated with acute PHM has been described as poor, and mortality is approximately ≥90% [4]. However, a difference in survival between MSE and generalized PHM has not been yet confirmed [4].

It is important to distinguish acute PHM from chronic PHM, which is commonly referred to as Lance–Adams syndrome in people. Presenting as a multifocal action myoclonus along with generalized myoclonus that appears days to weeks after the resolution of coma, chronic PHM is usually cortical in origin [5]. While it has not been yet reported in dogs and cats, in people, chronic PHM carries a more favorable prognosis, with patients usually regaining consciousness after several weeks [5].

Neurologic examination in both of our cases identified lesions neurolocalized to the forebrain and brainstem, areas where myoclonus can be generated in people [2, 3]. Both cases developed myoclonus while stuporous, and signs persisted in the days leading up to euthanasia. These clinical findings are consistent with a diagnosis of PHM. However, diagnosis of the origin of PHM was not confirmed in either case due to lack of a complete battery. In people, EEG and electromyography (EMG) are the gold standards for the definitive diagnosis of acute PHM and to distinguish between acute and chronic forms [16]. Preserved background reactivity on EEG carries a more favorable prognosis, whereas presence of any epileptiform activity, poly‐spike burst suppression, or subcortical myoclonus may suggest a poorer outcome [5]. Neither of our cases underwent EEG or EMG, which could have been helpful to confirm diagnosis and characterize the myoclonus. A limitation of the feline case is that GHIBI was not confirmed by magnetic resonance imaging (MRI) or postmortem examination, but was presumed due to the cat's history of CPA. However, in both cases, persistent myoclonus began within 24 h of CPA, the animals were stuporous in the presence of myoclonus, and myoclonus persisted over time despite administration of anticonvulsant medications. These clinical findings all make PHM likely in both cases.

A potential cause for prolonged neurologic recovery in Case 1 was weaning from mechanical ventilation and recovery from sedative drug administration. In an experimental study of healthy cats being mechanically ventilated, some took longer than 24 h to fully recover from sedation [17]. Sedation with ketamine was associated with a longer time to neurologic recovery compared with propofol alone or in combination with ketamine [17]. Human studies have found that different sedatives affect the prognosis for recovery from mechanical ventilation [18]. Benzodiazepine use has been associated with longer recovery time and time on mechanical ventilation in people compared with propofol or dexmedetomidine [19].

There are no current veterinary studies in clinically diseased patients assessing time to neurologic recovery from mechanical ventilation. If cases survive to hospital discharge, long‐term recovery is very good [20]. The cat in our case report was receiving midazolam at the time the myoclonus was noted, and then received dexmedetomidine and ketamine infusions for sedation, which may have impacted neurologic status and the perceived prognosis. There was initial improvement in the cat's mentation after recovery from sedation, followed by deterioration to being comatose with no improvement 48 h after weaning from mechanical ventilation. It seems unlikely that sedation was responsible for the ultimate deterioration in the cat's mental status since it improved initially after discontinuation of sedative drugs.

Acute PHM in people, especially generalized and when manifested in the first 72 h after CPA, has traditionally been considered a marker for poor prognosis [5, 13]. Acute PHM is usually transient, and affected patients generally either suffer deterioration (including death), continue to a chronic vegetative state, or improve with or without chronic PHM [13]. A randomized controlled trial in people again showed that acute PHM was often associated with poor outcome at 3 months follow‐up [21]. However, more recent studies suggested that acute PHM, while representing a sign of devastating anoxic–ischemic brain injury, does not always indicate irreversible brain damage and thus should not be associated with poor prognosis as a sole entity [22]. More specifically, if acute PHM is cortical with preserved EEG and a reactive background, it may respond to anticonvulsant treatment, and consequently, the outcome may become favorable [22].

The small number of cases and lack of EEG data in our case series preclude an assessment of the association of presumed PHM with prognosis. Both cases had persistent myoclonus until euthanasia. The persistent myoclonus, and the historically reported poor prognosis that accompanies it in people with PHM, may have introduced bias into discussions of prognosis with the owners. However, there were many factors involved in the decision in both cases to euthanize, largely due to finances and clinical deterioration. Case 1 had deterioration in mental status 24 h after weaning from mechanical ventilation, and Case 2 had deterioration of respiratory signs. Given the clinical deterioration of these cases, and lack of an evidence base in veterinary patients, it is possible that the myoclonus itself did not affect euthanasia decisions.

Limitations of this series include the lack of EEG (e.g., identification of possible epileptogenic discharges and association of the myoclonic episodes with discharges), leading to uncertainty whether these phenomena reflect an MSE or a subcortical myoclonus. Additionally, there were some dissimilarities between the case presentations seen here and acute PHM in people; specifically, in people, acute PHM is usually observed in comatose patients, whereas, in our cases, mentation was consistent with stupor. Therefore, any conclusive association should be made with caution, and the diagnoses in both of our cases are presumptive. Interestingly, lack of consensus on phenotypic and EEG features also exists in human neurology [23]. Further research is warranted for more precise characterization of the phenomenon. Another limitation of the series is that both animals were receiving sedative medications at the time the myoclonus was identified: Case 1, midazolam; Case 2, dexmedetomidine and trazodone. Therefore, iatrogenic induction of a depression to the mentation or drug‐induced myoclonus cannot be completely ruled out.

In conclusion, acute PHM is a neurologic sign likely of cortical or subcortical origin that may manifest within 48 h after CPA, CPR, and ROSC due to GHIBI, and may be seen in cats and dogs. Future larger studies in cats and dogs including a detailed clinical characterization of the myoclonus, in addition to MRI, EEG, and EMG, would be useful to determine whether the presence of acute PHM affects outcome.

## Author Contributions

**Theofanis Liatis**: conceptualization, investigation, writing – original draft, methodology, visualization, validation, writing – review and editing, project administration, resources. **Alison Robertson**: investigation, methodology, visualization, resources, writing – review and editing. **Alberta De Stefani**: methodology, writing – review and editing, supervision, resources.

## Conflicts of Interest

The authors declare no conflicts of interest.

## Supporting information




**Supporting File 1**: vec70140‐sup‐0001‐Video1.mp4

## Data Availability

The data that support the findings of this study are available from the corresponding author upon reasonable request.

## Endnotes

1 7.2% Sodium Chloride, Dechra Veterinary Products, Shropshire, UK.

2 20% Mannitol, Fresenius Kabi, Cheshire, UK.

3 Midazolam, Hameln Pharma Ltd, Gloucester, UK.

4 Vetivex 11 (Hartmanns) solution for infusion, Dechra Veterinary Products.

5 Potassium Chloride, Hameln Pharma Ltd.

6 Levetiracetam, UCB Pharma, Brussels, Belgium.

7 Ketamine, Dechra Veterinary Products.

8 Dexmedetomidine, Vetoquinol UK Ltd, Towcester, UK.

9 Propofol‐Lipuro Vet, Virbac, B Braun, Melsungen, Germany.

10 Mindray SV300 Pro ventilator, Mindray, China.

11 Lidocaine B. Braun, Melsungen.

12 Diltiazem, PilsCo Ltd, East Kilbride, UK.

13 Magnesium, Norbrook Laboratories Limited, Northern Ireland.

14 Amiodarone, Baxter Healthcare Corporation, IL.

15 Methadone, Animalcare Ltd, York, UK.

16 Beneheart D6 Defibrillator, Mindray, China.

17 Flumazenil, Hameln Pharma Ltd.

18 Naloxone, B Braun.

19 Atropine, Hameln Pharma Ltd.

20 IDEXX SNAP 4Dx Plus Test ELISA snap test, IDEXX, Hoofddorp, The Netherlands.

21 Dexamethasone, MSD Animal Health, Milton Keynes, UK.

22 Sotalol, Bristol Laboratories Ltd, Herts, UK.

23 Trazodone, Manx Healthcare Ltd, Warwick, UK.

24 Vapotherm High Flow Nasal Oxygen Therapy, Vapotherm Inc., Exeter, NH.

25 Amoxicillin‐clavulanic acid, Bowmed Ibisqus Limited, Wrexham, UK.
